# Ataxia Telangiectasia-Mutated (ATM) Kinase Activity Is Regulated by ATP-driven Conformational Changes in the Mre11/Rad50/Nbs1 (MRN) Complex*[Fn FN2]

**DOI:** 10.1074/jbc.M113.460378

**Published:** 2013-03-22

**Authors:** Ji-Hoon Lee, Michael R. Mand, Rajashree A. Deshpande, Eri Kinoshita, Soo-Hyun Yang, Claire Wyman, Tanya T. Paull

**Affiliations:** From the ‡ Howard Hughes Medical Institute and the Department of Molecular Genetics and Microbiology, the Institute for Cellular and Molecular Biology, The University of Texas at Austin, Austin, Texas 78712 and; the Departments of § Genetics and; ¶ Radiation Oncology, Erasmus University Medical Center, PO Box 2040, 3000 CA Rotterdam, The Netherlands

**Keywords:** DNA Repair, Phosphorylation, Phosphorylation Enzymes, Protein Conformation, Protein DNA-Interaction

## Abstract

The Ataxia Telangiectasia-Mutated (ATM) protein kinase is recruited to sites of double-strand DNA breaks by the Mre11/Rad50/Nbs1 (MRN) complex, which also facilitates ATM monomerization and activation. MRN exists in at least two distinct conformational states, dependent on ATP binding and hydrolysis by the Rad50 protein. Here we use an ATP analog-sensitive form of ATM to determine that ATP binding, but not hydrolysis, by Rad50 is essential for MRN stimulation of ATM. Mre11 nuclease activity is dispensable, although some mutations in the Mre11 catalytic domain block ATM activation independent of nuclease function, as does the mirin compound. The coiled-coil domains of Rad50 are important for the DNA binding ability of MRN and are essential for ATM activation, but loss of the zinc hook connection can be substituted by higher levels of the complex. Nbs1 binds to the “closed” form of the MR complex, promoted by the zinc hook and by ATP binding. Thus the primary role of the hook is to tether Rad50 monomers together, promoting the association of the Rad50 catalytic domains into a form that binds ATP and also binds Nbs1. Collectively, these results show that the ATP-bound form of MRN is the critical conformation for ATM activation.

## Teaser

**Background:** The Mre11/Rad50/Nbs1 (MRN) complex regulates DNA repair and signaling through the Ataxia Telangiectasia-Mutated (ATM) kinase.

**Results:** ATM activation requires ATP binding by Rad50 and the coiled-coils but not ATP hydrolysis, zinc hook connection, or Mre11 nuclease function.

**Conclusion:** The ATP-bound form of MRN with Rad50 catalytic domains engaged is the form that activates ATM.

**Significance:** ATP-driven changes in MRN conformation control ATM signaling.

## Introduction

The ATM protein kinase plays a central role in signaling the presence of DNA double-strand breaks (DSBs)^3^ in eukaryotic cells. Activation of ATM occurs very rapidly, within a few minutes of DNA break incidence (1), and is dependent on the Mre11/Rad50/Nbs1 (MRN) complex for its recruitment to sites of DNA damage (2). MRN also functions to promote a transition in ATM conformation from an inactive dimeric form to an active monomeric form, and increases the affinity of ATM for its substrates (3).

**FIGURE 1.**
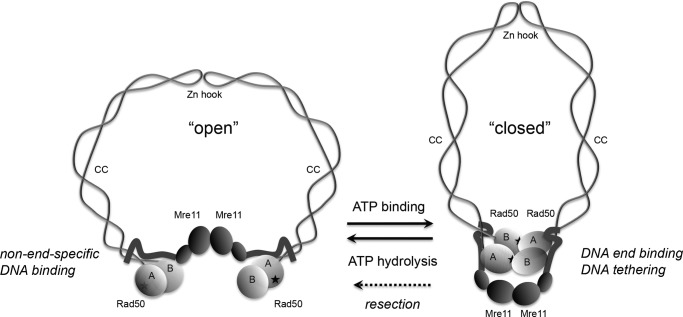
**Schematic model of MRN in the absence of ATP (*open*) or the presence of ATP (*closed*), based on structural analysis of bacterial and archaea Mre11/Rad50 complexes** (6, 8). Rad50 is shown as light gray globular domains containing Walker A (*A*) and Walker B (*B*) motifs connected by intramolecular, antiparallel coiled-coils (*CC*), that attach to each other through the zinc hook motifs (*Zn hook*). *Stars* indicate the approximate positions of the ATP-binding residues in Rad50. Mre11 is shown as *dark gray* globular domains representing the nuclease/dimerization domain and the capping domain, connected to the linker that binds to the coiled-coils of Rad50. The closed state is associated with a higher affinity for DNA ends and with DNA tethering, while the movement from the closed to the open state is required for Mre11 nuclease activity and promotion of resection.^5^ The zinc hook is shown attached here, but the complex has also been observed with hooks unattached, both in solution and bound to DNA (30).

The MRN complex is a large, multifunctional protein assembly that possesses endo-and exonucleolytic activity through the Mre11 component and ATP binding, hydrolysis, and adenylate kinase activity through the Rad50 component. Mre11/Rad50 (MR) complexes in prokaryotes and MRN complexes in eukaryotes are important for DNA double-strand break recognition and repair (4). The eukaryotic forms are also essential for meiotic DSB processing and telomere maintenance, as well as for signaling through the ATM kinase (5). Rad50 has an overall domain organization that is similar to the family of Structural Maintenance of Chromosomes (SMC) proteins which regulate chromatid cohesion and chromosome condensation. The Walker A and Walker B ATP-binding domains in each Rad50 protein associate together through an antiparallel association of the coiled-coil domains (Fig. 1). Each Rad50 protein binds to another through ATP-dependent association of the Walker A/B domains into a homodimer, and also through another association at the apex of the coiled-coils at the zinc hook domain. In addition, a dimer of Mre11 also links the ATP-binding domains of Rad50 through an association at the base of the coiled-coils.

Recent structural analysis of Mre11/Rad50 proteins from bacteria and archaea showed that the ATP-binding domains of Rad50 bound to Mre11 are in an open configuration in the absence of ATP (6). In contrast, the ATP-bound form of the complex is a closed configuration with the Mre11 nuclease domains in a dimer underneath the Rad50 head domains (7, 8). These results suggest that Mre11/Rad50 complexes are in a dramatically different conformation when bound to ATP compared with the nucleotide-free conformation (Fig. 1).

To better understand how these dynamic, ATP-driven changes in the MRN complex affect the regulation of ATM kinase activity, we employ here an analog-sensitive version of ATM to separate the nucleotide pools used by the kinase and by MRN. In addition, we investigate the role of Mre11 nuclease activity and the Rad50 coiled-coil domain in ATM activation. These experiments show that the “closed,” ATP-bound state of MRN is essential for ATM stimulation, thus explaining why it is important that MRN complexes have a slow rate of hydrolysis. Lastly, we show that Nbs1 binds to MR preferentially when it is in the “closed” state and suggest that the hook domain in Rad50 primarily serves to increase the local concentration of the Rad50 head domains, making Rad50 head dimerization independent of MRN concentration.

## EXPERIMENTAL PROCEDURES

### Protein Expression

Wild-type MRN, M(H129N)RN, M(H129L/D130V)RN, and M(D130V)RN complexes were expressed in Sf21 insect cells by coexpression with baculovirus prepared from the transfer vectors pTP11 (wild-type Rad50), pTP1118 (C2G Rad50), pTP813 (wild-type Mre11), pTP1441 (Mre11(H129N)), pTP2196 (Mre11(H129L/D130V)), pTP2411 (Mre11(D130V), and pTP288 (wild-type Nbs1) as described previously (Bhaskara *et al.*, 11; Paull & Gellert, 14; Paull & Gellert, 18). The H129N, H129L/D130V, and D130V versions of Mre11 and the C2G (C681G/C684G) version of Rad50 were generated by QuikChange mutagenesis (Stratagene) from pTP813 and pTP11, respectively (sequences available upon request). To generate the DelCC version of Rad50, the N terminus (amino acids 1–216) and C terminus (amino acids 1104–1312) of Rad50 were amplified and a linker peptide was included between the domains (PPAAAGG). The gene was cloned into pFastBacHtB (Invitrogen) to create pTP516. Rad50 coiled coils (amino acids 203–1117) were amplified and cloned in pFastbacHTA to generate pTP1160. To generate Rad50 (FKBP-A), the N terminus of Rad50 (amino acids 1–675) was amplified from pTP11 and fused to a derivative of FKBP12 amplified from pC_4_-Fv1E (Ariad Pharmaceuticals) to create pTP1326. To generate Rad50 (FKBP-B), the C terminus of Rad50 (amino acids 686–1312) was amplified from pTP11 with a start codon at a.a. 686 and re-ligated into pTP11 to create pTP1327. pTP11, pTP1118, pTP1326, and pTP1327 were directly transfected into Sf21 cells with linearized baculovirus DNA (BD Pharmingen) to make recombinant baculovirus, whereas the other transfer vectors were converted into bacmids pTP814 (wild-type Mre11), pTP1443 (Mre11(H129N)), pTP2197 (Mre11(H129L/D130V)), pTP2412 (Mre11(D130V), pTP291 (wild-type Nbs1), pTP 1161 (Rad50 coiled coils), and pTP543 (Rad50 DelCC) and were used to make virus according to manufacturer instructions for the Bac-to-Bac system (Invitrogen). Expression constructs for Flag-tagged wild-type and HA-tagged ATM were gifts from M. Kastan and R. Abraham, respectively. Y2755A ATM was generated using Quikchange XL site-directed mutagenesis (Stratagene) from wild-type ATM pcDNA3 expression plasmid (sequences available upon request). Cesium-purified plasmid DNA was used to transfect human 293T cells and express recombinant ATM as described previously (9). The *Escherichia coli* expression constructs for GST-p53 and GST-Chk2 were described previously (10).

### Protein Purification

Wild type and mutant MRN were purified as described (11). Rad50 coiled coils were purified similar to wild-type MRN as described (9). Dimeric ATM was made by transient transfection of expression constructs into 293T cells using calcium phosphate and purified as described previously (9). GST-p53 protein was expressed and purified in *E. coli* as described previously (10). Protein concentrations were determined by quantification of protein preparations with standards on colloidal Coomassie-stained SDS-PAGE gels using the Odyssey system (LiCor).

### ATM Kinase Assays in Vitro

**FIGURE 2.**
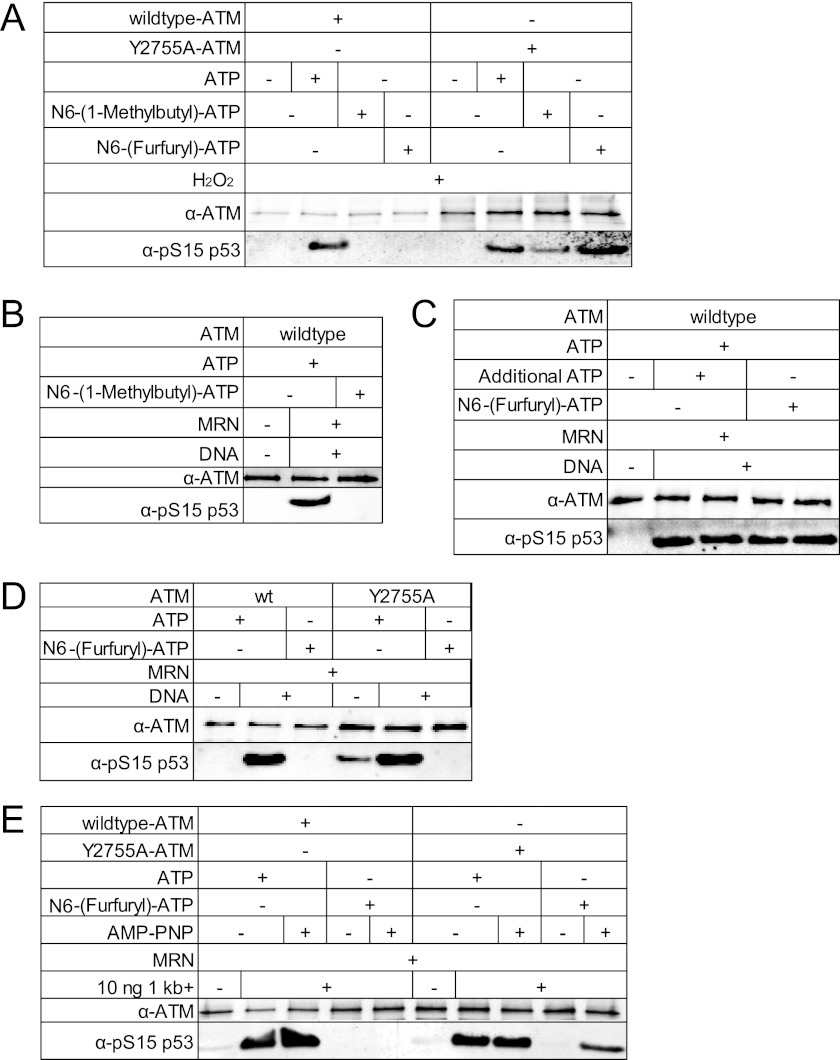
**MRN stimulation of ATM activity requires ATP binding but not hydrolysis.** *A*, kinase assays were performed with wild-type or Y2755A ATM and either ATP or the ATP analogs *N*6(1-methylbutyl)-ATP or *N*6(1-furfuryl)-ATP as indicated, using H_2_O_2_ to activate ATM in the absence of MRN. Recombinant GST-p53(1–100) was used as the substrate, and phosphorylation of p53 Ser-15 was assessed by Western blotting and a phospho-specific antibody directed against Ser-15. *B*, MRN and DNA were used to activate wild-type ATM in the presence of ATP and *N*6(1-methylbutyl)-ATP as indicated. Kinase activity was assessed as in *A. C*, kinase assays were performed as in *B* except with *N*6(1-furfuryl)-ATP as indicated. *D*, wild-type or Y2755A ATM was activated by DNA and MRN in the presence of either ATP or *N*6(1-furfuryl)-ATP as indicated. *E*, MRN/DNA was used to activate wild-type or Y2755A ATM with ATP or *N*6(1-furfuryl)-ATP in the presence or absence of the non-hydrolyzable ATP analog AMP-PNP.

ATM kinase assays with MRN and DNA were performed with 0.2 nm dimeric ATM, 50 nm GST-p53 substrate, 4.8 nm MRN, and ∼140 nm linear double-stranded DNA as indicated in the figure legends. Kinase assays were performed in kinase buffer (50 mm HEPES, pH 7.5, 50 mm potassium chloride, 5 mm magnesium chloride, 10% glycerol, 1 mm ATP, and 1 mm DTT) for 90 min at 30 °C in a volume of 40 μl as described previously (3). Kinase assays with oxidation were performed in the absence of DTP with 2.7 mm H_2_O_2_ (Fig. 2*A*). The ATP analogs *N*6-(1-methylbutyl)adenosine-5′-*O*- triphosphate and 10 mm *N*6-furfuryladenosine-5′-*O*-triphosphate were obtained from Biolog (BLG-M027-05 and BLG-F007-05, respectively) and used at a final concentration of 1 mm. Phosphorylated p53 (Ser-15) was detected as described previously (3) using phospho-specific antibody from Calbiochem (PC461). ATM was detected with mouse anti-ATM (GeneTex; GTX70103). Each experiment was performed several times, and a representative example is shown in the figure.

### DNA Binding and Nuclease Assays

Gel mobility shift assays with ^32^P-labeled TP423/TP424 (containing 15 nt and 16 nt 3′ overhangs on the ends of a 34 bp duplex) were performed as previously described (supplemental Fig. S2) (12). Gel shift assays shown in Fig. 6*A* were performed with a 41 bp double-stranded oligonucleotide substrate composed of TP2151 (5′-CTTGCATGCCTCAGCTATTCCGGATTATTCATACCGTCCCA-3′) annealed to TP2109 (5′-TGGGACGGTATGAATAATCCGGAATAGCTGAGGCATGCAAGTCTA-3′), with TP2109 labeled at the 3′-end with Cy5 at the end of a 4-nt 3′ overhang. Reactions contained 10 nm DNA substrate with 25 mm MOPS, pH 7.0, 20 mm Tris, pH 8.0, 5 mm MgCl_2_, 8% glycerol, 80 mm NaCl, 2 mm dithiothreitol (DTT), and 0.5 mm AMP-PNP. Binding assays with the coiled-coils were similar except performed with a 249 bp internally labeled DNA was made by PCR, gel purified, and used in reactions as above, which were separated in a 0.7% agarose gel in 89 mm Bis-Tris-Borate buffer as described previously (13). Nuclease assays with ^32^P-labeled TP74/TP124 (containing a 4-nt 5′ overhang on a 46-bp duplex) were performed as previously described (14).

### Gel Filtration

A total of 100 nm MR(FKBP) was incubated with 200 nm Nbs1 in the presence of 100 nm AP20187 (Ariad Pharmaceuticals) at 30 °C for 1 h in final volume of 60 μl and then the samples were directly loaded on a Superose 6 PC 3.2/30 gel filtration column (GE) equilibrated in buffer A (25 mm Tris, pH 8.0, 100 mm NaCl, 10% glycerol, 1 mm DTT). Samples from the 50-μl fractions were separated by SDS-PAGE and analyzed by Western blotting with antibodies directed against Rad50 (GTX70228, Genetex) and Nbs1 (GTX70224, Genetex), followed by detection with IRdye 800 anti-mouse (Rockland, 610-132) secondary antibodies. Western blots were analyzed and quantitated using a Licor Odyssey system.

### Scanning Force Microscopy (SFM)

#### 

##### DNA Substrates

The 3-kb linear dsDNA was produced by digestion of pUC19 plasmid with EcoRI. This resulting linear DNA was subsequently purified by standard phenol extraction and ethanol precipitation. Imaging: for images of protein alone, 3 nm human MR (wild-type or indicated mutants, was incubated for 1 min in 20 μl of protein storage buffer (25 mm Tris-HCl, pH 8.0, 10% glycerol, 100 mm NaCl, 1 mm DTT. For imaging, reactions were diluted 5-fold by adding 80 μl of deposition buffer (10 mm HEPES (pH 8.0), 20 mm MgCl_2_) and deposited onto a freshly cleaved mica surface for 1 min.

DNA-Protein complexes for SFM imaging were formed by first incubating equimolar amounts of NBS1 and MR together for 1 min to reconstitute ∼3 nm MRN in 18 μl of protein storage buffer. Then 2 μl of 5 nm 3-kb linear dsDNA was added to make a final concentration of 0.5 nm 3-kb linear dsDNA and incubated for another minute. For binding reactions in the presence of ATP, 2 μl of 10 mm ATP, and 2 μl of 5 nm 3-kb linear dsDNA were added to make final concentrations of 1 mm ATP and 0.5 nm 3-kb linear dsDNA. For imaging, reactions were diluted 5-fold by adding 80 μl of deposition buffer and deposited onto a freshly cleaved mica surface for 1 min.

For all samples, after absorption onto freshly cleaved mica for 1 min, the mica was rinsed three times with MilliQ water and dried with filtered air. Samples were imaged in air at room temperature and humidity by tapping mode SFM using Nanoscope IV (Digital Instruments). Silicon Nanotips were from Digital Instruments (Nanoprobes). Images were collected at 2.5 μm × 2.5 μm and processed only by flattening to remove background slope.

### KinExA Measurements

KinExA measurements of MR-Nbs1 affinity was performed by attaching Nbs1 to beads and using these to capture unbound MR complexes. To prepare Nbs1-bound beads, 30 μg of Nbs1 was mixed in 1 ml of PBS buffer and incubated with the PMMA beads (440176, Sapidyne) on a rotator for 2 h at room temperature. Supernatant was removed after precipitating the beads by sitting the tube in the rack for 1 min and 1 ml of PBS buffer containing 10 mg/ml BSA was added to the tube. After 1-h incubation at room temperature, the mixture of Nbs1-bound PMMA beads with PBS buffer were added to the Autosampler vial for KinExA (A12116, Sapidyne) containing 27 ml of PBS buffer and kept at 4 °C until use. To prepare MR samples, 25 nm, 5 nm, and 1 nm wild-type or mutant MR complex was incubated with 6.1 pm to 400 nm Nbs1 in 2-fold dilutions for 1 h at room temperature. Each sample was passed through the column charged with Nbs1-bound beads at 0.25 ml/min. The volume of MR samples used were varied: 0.25 ml for 25 nm MR; 1.25 ml for 5 nm MR; 7.25 ml for 1 nm MR. After washing the column with 0.125 ml PBS, 0.5 ml primary antibody solution containing 1 μg/ml of anti-Mre11 antibody (GTX70212, Genetex) and 1 mg/ml BSA in PBS were flowed through the column. After another wash with PBS, 0.5 ml of secondary antibody solution containing 0.5 μg/ml of Cy5-conjugated anti-mouse IgG (A10524, Invitrogen) and 1 mg/ml BSA in PBS buffer were flowed through the column. Cy5 signals were detected by KinExA 3200 (Sapidyne) and *K_d_* were calculated with n-Curve Analysis (Sapidyne).

## RESULTS

### 

#### 

##### MRN Requires ATP Binding but Not Hydrolysis for ATM Activation

Both the MRN and ATM complexes utilize ATP, which cannot be removed from an *in vitro* kinase assay since the activity of ATM can only be assessed by monitoring ATP-dependent phosphorylation. To specifically manipulate the nucleotide pool used by ATM, we generated a mutant form of the kinase which allows the utilization of *N*6-substituted-ATP analogs by generating a cavity within the ATP-binding pocket (15). Mutation of tyrosine 2755 in the ATP-binding pocket of ATM was previously shown to allow the kinase to accept *N*6-modified ATP analogs (16). The Y2755A mutant form of ATM was expressed and purified as previously described (9) (supplemental Fig. S1), and tested *in vitro* in a kinase assay (Fig. 2*A*). In this experiment, hydrogen peroxide was used to activate ATM (17), to assess the ability of ATM to utilize ATP analogs in the absence of the MRN complex. As shown previously, *N*6-(1-methylbutyl)-ATP was utilized by the Y2755A mutant ATM, and was not utilized by the wild-type enzyme (Fig. 2*A*), as measured by phosphorylation of a GST-p53 substrate and phospho-specific p53 antibody in a quantitative Western blot as described previously (3). However, further experiments showed that *N*6-(1-methylbutyl)-ATP inhibited the activation of wild-type ATM by MRN/DNA, even in the presence of ATP (Fig. 2*B*). This effect was specific to reactions containing MRN, so we conclude that *N*6-(1-methylbutyl)-ATP is toxic to MRN function. We also found that several other *N*6-modified ATP analogs were similarly toxic,^4^ with the exception of *N*6-(furfuryl)-ATP. This nucleotide was utilized by Y2755A ATM but not by wild-type ATM when activated by oxidation (Fig. 2*A*) and also does not prevent MRN/DNA activation of wild-type ATM in the presence of ATP (Fig. 2*C*).

The Y2755A mutant ATM protein was tested in an MRN/DNA-dependent assay, and was found to be unable to phosphorylate p53 in the presence of *N*6-(furfuryl)-ATP alone, demonstrating that MRN requires ATP to activate ATM (Fig. 2*D*). The Y2755A mutant also shows some DNA-independent activation that is only observed with ATP, not with the *N*6-(furfuryl)-ATP analog. To determine if ATP hydrolysis is required by MRN, the same reaction was performed in the presence of the nonhydrolyzable ATP analog AMP-PNP (Fig. 2*E*). Notably, Y2755A ATM phosphorylated p53 in the presence of MRN/DNA, *N*6-(furfuryl)-ATP, and AMP-PNP while wild-type ATM was inactive under these conditions. Taken together, these results show that MRN requires ATP for ATM activation but that hydrolysis is not essential.

##### Mre11 Nuclease Activity Is Not Essential for ATM Kinase Activity

The Mre11 component of the MRN complex exhibits manganese-dependent 3′ to 5′ exonuclease and endonuclease activity *in vitro* (14, 18). In the presence of magnesium ions (in the absence of manganese), MR complexes show a weaker activity that is primarily endonucleolytic (19, 20). Mre11-dependent nuclease activity has been shown genetically to be required for DNA end processing in meiosis at Spo11-generated break sites in budding yeast, but is not required for processing of DNA ends generated by site-specific endonucleases in vegetatively growing cells (21). Mre11 nuclease activity was implicated in the production of small oligonucleotide products in *Xenopus* egg extracts, which were suggested to be required for ATM activation in this *in vitro* system (22). In contrast, mouse embryonic fibroblasts derived from an embryo expressing a nuclease-deficient Mre11 (H129N) were shown to exhibit normal ATM activation, although the embryos expressing this allele showed early lethality (23).

**FIGURE 3.**
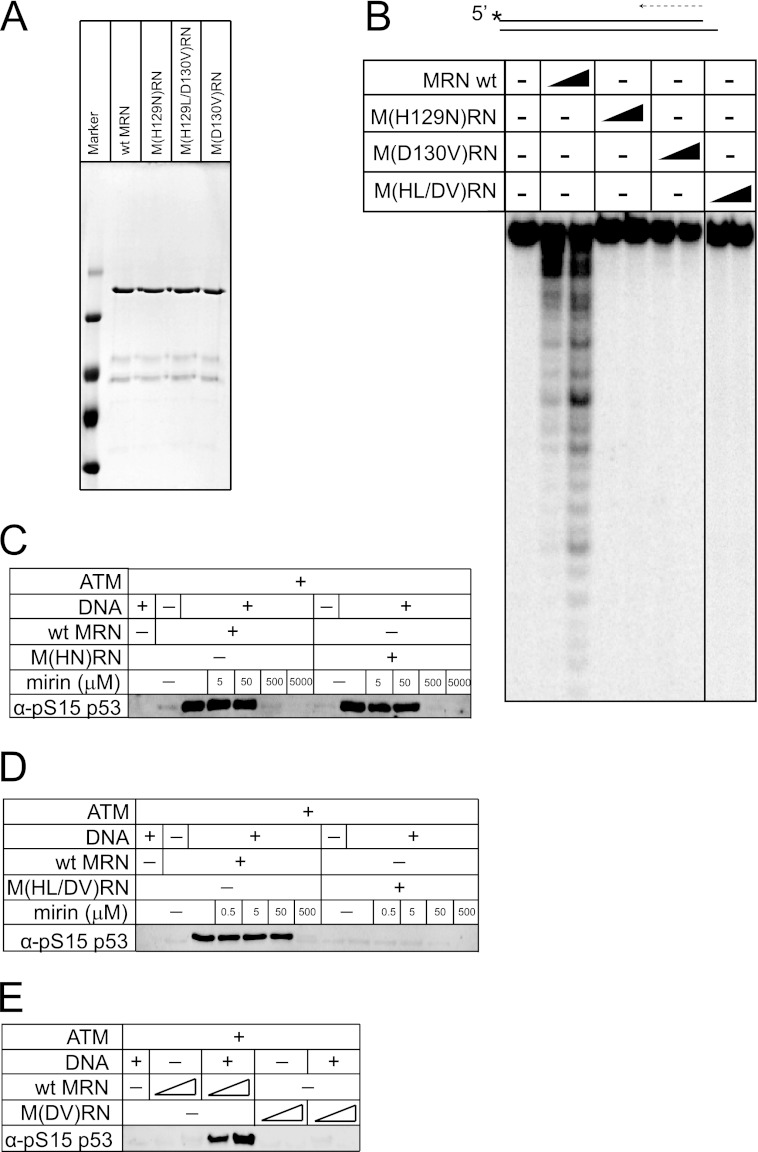
**Mre11 nuclease activity is not required for ATM activation.** *A*, SDS-PAGE of wild-type and mutant MRN complexes, stained with Coomassie Blue. *B*, nuclease assays of wild-type MRN (*wt*), M(H129N)RN, M(D130V)RN, and M(H129L/D130V)RN in manganese chloride using a ^32^P-labeled double-stranded oligonucleotide substrate as indicated. Concentrations of each complex were 10, 20, and 40 nm, respectively. Reaction products were separated with a denaturing polyacrylamide sequencing gel and analyzed by phosphorimager. *C*, ATM kinase assay as in Fig. 2*B* except with wild-type MRN or M(H129N)RN complexes. Mirin was included at 5, 50, 500, or 5000 μm as indicated. *D*, kinase assays as in *C* except with the M(H129L/D130V)RN complex. *E*, kinase assay as in *C* except with the M(D130V)RN complex.

To investigate whether Mre11 nuclease activity is required for ATM activation, we expressed and purified two forms of human MRN containing mutations in the Mre11 nuclease domain similar to mutants previously described in yeast (21, 24)(Fig. 3*A*). Human Mre11(H129N) is equivalent to yeast Mre11(H125N) which was previously shown to be nuclease-deficient *in vitro* (21). Mre11(H129L/D130V) is equivalent to the yeast *mre11-3* allele that contains a mutation in the same conserved histidine residue as well as the adjacent aspartate residue (24). Budding yeast strains expressing these mutants exhibit ionizing radiation sensitivity that is intermediate between a wild-type strain and an *mre11* deletion strain (21, 24). As shown in Fig. 3*B*, both the M(HN)RN and the M(HL/DV)RN human complexes appear to be completely deficient in nuclease activity compared with wild-type MRN under conditions where nuclease activity is most clearly visible (*i.e.* a 5′ ^32^P-labeled substrate in manganese chloride). Both complexes bind to DNA in gel mobility shift assays with AMP-PNP similarly to the wild-type complex (supplemental Fig. S2), demonstrating that the defect in nuclease activity is not related to substrate binding. As shown in Fig. 3, *C* and *D*, the M(HN)RN complex promotes ATM activity as efficiently as wild-type MRN, yet the M(HL/DV)RN complex fails to activate ATM. The difference between the activity of these two mutant complexes is likely due to the D130V mutation, rather than a difference in nuclease activity, since the single mutant M(DV)RN complex was also completely deficient in activating ATM (Fig. 3*E*). The M(DV)RN complex failed to show exonuclease activity *in vitro*, similar to the other mutants (Fig. 3*B*). We conclude from this set of experiments that Mre11 nuclease activity is not essential for ATM activation, as evidenced by the full activity of the M(HN)RN complex, although mutations in the Mre11 catalytic domain can have strong effects on this process, independent of nuclease function.

The mirin compound was first isolated in a chemical screen for compounds that block MRN/ATM-mediated responses to DSBs in *Xenopus* egg extracts (25). Mirin was shown to block Mre11 exonuclease activity and MRN-dependent ATM activation *in vitro*, and to inhibit the ionizing radiation-induced G_2_/M checkpoint and homologous recombination in mammalian cells. Here we show that mirin inhibition of ATM activation is independent of Mre11 nuclease inhibition since the M(HN)RN and M(HL/DV)RN mutant complexes are inhibited equivalently to the wild-type enzyme despite the fact that they are nuclease-deficient (Fig. 3, *C* and *D*). The effects of mirin have been ascribed to its effects on Mre11 nuclease activity, but as we show here, mirin is inhibitory of MRN function independent of the nuclease activity of the complex.

##### The Rad50 Coiled-coils Are Essential for ATM Activation

**FIGURE 4.**
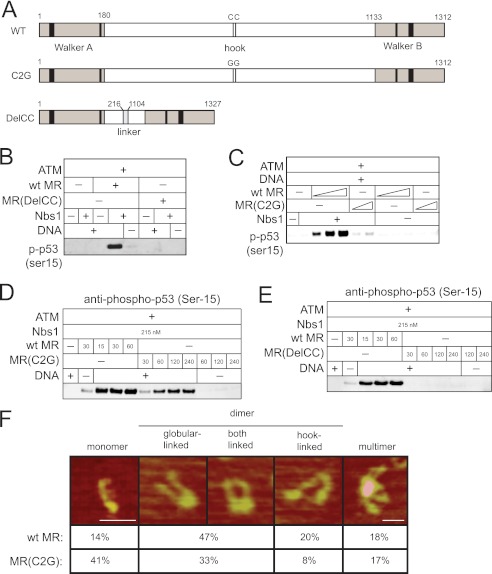
**The Rad50 coiled-coil domains are important for ATM activation.** *A*, schematic diagram of Rad50 including the catalytic domains (Walker A and Walker B) and the cysteines in the zinc hook. The C2G and DelCC mutant forms are shown. *B*, ATM kinase assay as in Fig. 3*C* but with 0.2 nm dimeric ATM, 30 nm MR, 215 nm Nbs1, 50 nm GST-p53 substrate, and 10 ng linear DNA, probed with antibody directed against phosphoserine 15 of p53, Mre11, or Rad50 as indicated. *C*, kinase assays as in *B* except with varying MR concentrations (15, 30, or 60 nm for wild-type MR; 30 and 60 nm for MR(C2G)). *D* and *E*, kinase assays in *B* except with varying MR concentrations indicated. *F*, SFM images of wild-type MR are shown, depicting different categories of complexes shown. Scale bar, 50 nm. Color indicates height (dark to bright, 0–3 nm). Percentages of monomer, dimer, or multimer forms of wild-type MR and MR(C2G) were calculated from SFM images (*n* = 217 wild-type; *n* = 209 C2G). The globular linked dimers include those linked at both ends. These were not separately counted and are usually about 50% linked by globular domain only and 50% linked at both globular and hook domains.

The nucleotide-binding domains of Rad50 are separated by long coiled-coil regions that fold in an antiparallel manner (see Fig. 1). The apex of the coiled-coil region contains a zinc-binding domain (the zinc hook) that has previously been shown to mediate a non-covalent association with another Rad50 molecule (26). To determine the importance of the coiled-coils and the zinc hook for ATM activation, mutants were made in which the cysteines that mediate zinc interaction were mutated (Rad50 C2G), or the entire coiled-coil region was deleted and replaced with a short flexible linker (Rad50 DelCC)(Fig. 4*A*). A comparison of these mutant complexes with wild-type MRN in the ATM kinase assay indicated that neither complex stimulated ATM activity (Fig. 4, *B* and *C*), although higher levels of the C2G mutant were able to restore the ability of ATM to phosphorylate substrate (Fig. 4*D*). In contrast, similarly high levels of the DelCC MRN mutant could not activate ATM (Fig. 4*E*), indicating that the coils are essential for MRN stimulation of ATM.

To determine if the MR(C2G) mutant has the expected architecture and if association between the Rad50 coiled-coil domains was altered, we examined the wild-type and C2G forms of the MR complex using SFM (Fig. 4*F*). The wild-type MR complex typically appears in several forms, defined by the number of Rad50 coiled-coils that can be identified and their arrangement (27). The most prominent forms are dimers and include two Rad50 and two Mre11, with the Rad50 coiled coils emanating from a globular domain and often also associated via the Zn hooks at their other end. Less frequent are monomers, having only one Rad50 and dimers only associated via Zn hook interactions, and multimers identified as having 4 or more Rad50's associated via a common globular domain. Consistent with the expected loss of zinc hook function, the C2G mutant preparation contained higher levels of monomer species and fewer dimeric species, with the majority of the dimers appearing to be linked through the catalytic head domains. The difference between the number of monomers observed with wild-type *versus* Rad50 C2G was statistically significant (*p* < 0.001 by chi square test), as was the difference between wild-type and Rad50 C2G with respect to the number of head-linked dimers (*p* < 0.001 by chi square test).

**FIGURE 5.**
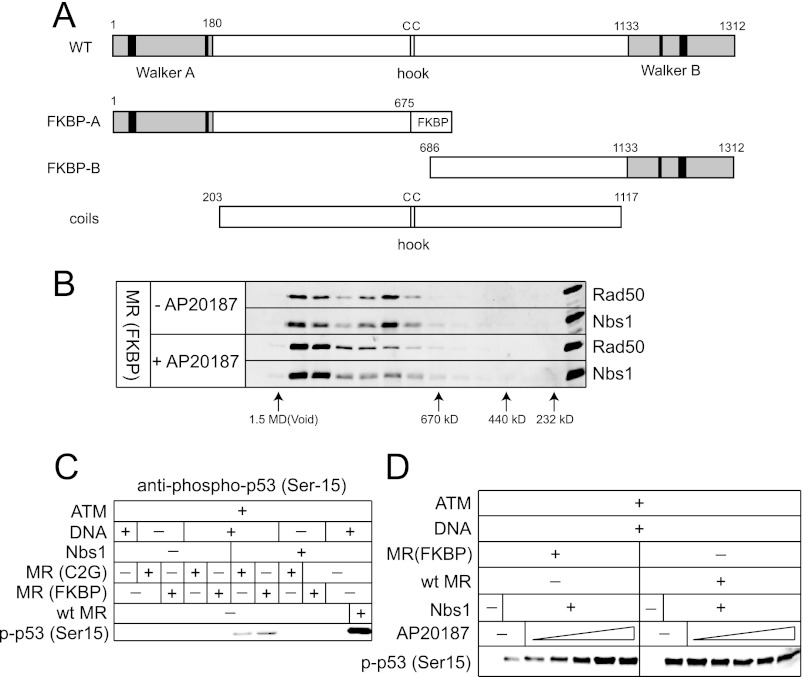
**Rad50 dimerization is essential for ATM activation.** *A*, schematic diagram of wild-type Rad50, mutant Rad50 containing the homodimerization domain FKBP replacing the zinc hook domain, and the coiled-coils deletion mutant. *B*, elution profiles of Rad50 (FKBP) and Nbs, in the presence or absence of the rapamycin analog AP20187 (100 nm) after incubation of MR (FKBP) and Nbs1, by gel filtration. Fractions from the Superose 6 separations were analyzed by SDS-PAGE and analyzed by Western blotting using antibodies directed against Rad50 and Nbs1. *C*, kinase assays as in Fig. 3*C* except with 30 nm wild-type MR, MR(C2G), and MR(FKBP). *D*, kinase assays as in *C* but with varying amounts of AP20187 (25, 50, 100, 200, and 400 nm).

Considering the ability of the C2G mutant MRN complex to stimulate ATM (albeit requiring higher concentrations of protein), we asked whether a heterologous homodimerization domain would substitute for the zinc hook in ATM activation. We created complexes containing an FKBP homodimerization domain substituted for the zinc hook region in the N-terminal half of the Rad50 protein and coexpressed this mutant with the C-terminal half of Rad50 in a separate polypeptide (Fig. 5*A*). After purification, this complex migrated in gel filtration as a mixture of two different species but the addition of the rapamycin analog AP20187 induced a shift to the larger species, indicative of dimerization of two Rad50 split complexes (Fig. 5*B*). In the ATM kinase assay, the MR(FKBP) mutant stimulated ATM poorly compared with the wild-type MR, very similar to the result with the MR(C2G) mutant, but addition of AP20187 restored ATM activity to normal levels (Fig. 5, *C* and *D*). A heterologous connection between Rad50 molecules is thus sufficient for ATM stimulation, similar to previous findings for Rad50 function in budding yeast (28).

##### The Rad50 Coiled-coil Domain Is Important for MRN DNA Binding

**FIGURE 6.**
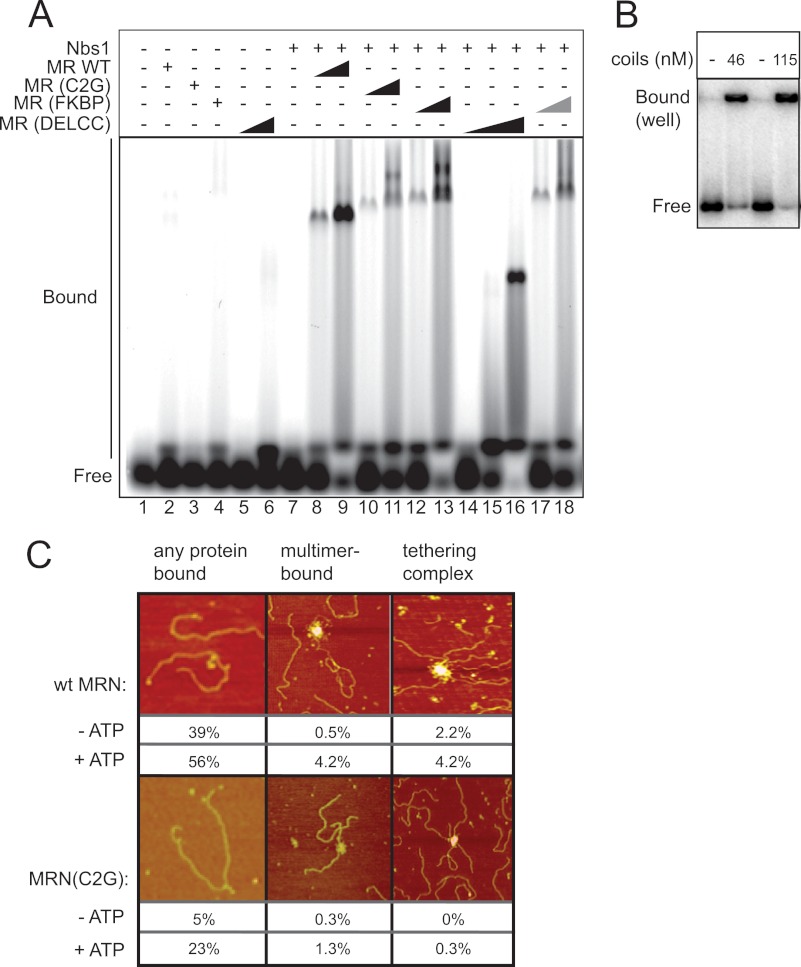
**DNA binding by hMRN complexes.** *A*, gel mobility shift assay with 10 nm Cy5-labeled 41 bp dsDNA substrate using 100 nm and 200 nm MR wild-type, C2G, and FKBP complexes and 100, 200, and 800 nm MR(DelCC) complex. Equimolar amounts of Nbs1 were added where indicated. MR(FKBP) reactions in lanes 17 and 18 also contained 200 nm AP20187. *B*, gel shift assay as in *A* with a 249 bp ^32^P-labeled DNA and either 46 or 115 nm Rad50 coiled-coil domain (residues 203 to 1117). *C*, SFM images showing DNA binding and DNA tethering by hMRN complexes. Different types of DNA binding events observed were calculated (*n*, 183 wild-type (−ATP); *n*, 119 wild-type (+ATP); *n*, 306 C2G (−ATP); *n*, 375 C2G (+ATP)). The images of monomer/dimer are ∼750 nm square; all others are ∼1.5–1.7 micrometer square. Color indicates height (dark to bright, 0–3 nm). The quantification of the “any protein bound” category includes monomer/dimer bound (as shown) as well as multimer-bound and tethering complexes. *Multimers*, larger than 4 Mre11/Rad50 molecules in a single complex; *tethering complex*, a complex bound to more than one DNA molecule.

The comparison between the DelCC, C2G, and FKBP Rad50 mutants suggested that the hook domain was important, but any means of connecting Rad50 coiled-coils would suffice. In contrast, the coils were essential for ATM activation since the DelCC mutant was inactive at all concentrations tested. To determine if the absence of the coiled-coil domain affects the DNA binding properties of MRN, we compared all the mutants in a gel mobility shift assay (Fig. 6*A*). In the presence of Nbs1, wild-type full-length MR forms a large complex that is dependent on ATP (or AMP-PNP) (12). Both of the mutants containing coiled-coils (C2G, FKBP) formed complexes in this assay similar in mobility to the wild-type complex, but the DelCC complex failed to bind DNA in the 100 to 200 nm protein concentration range. Only with much higher levels of DelCC MR (800 nm) was a complex observed in the gel (Fig. 6*A*, *lane 16*), demonstrating that the coils are important for DNA binding. To pursue this idea further, the coils were expressed separately from the Rad50 head domains (see Fig. 5*A*) and tested for DNA binding (Fig. 6*B*). Surprisingly, the coils alone exhibited a remarkable DNA binding ability that was extremely cooperative.

Although the MR(C2G) appeared to bind to DNA in the gel shift assay, the altered mobility of the complexes in the gel compared with the wild-type complex and the efficiency of complex formation suggested that the MR(C2G) mutant has a deficiency in DNA binding. Possible differences in the arrangement of protein bound to DNA were investigated by SFM imaging of complexes formed by MR(C2G)N and wild-type MRN and DNA, summarized in Fig. 6*C*. Several different classes of protein-DNA complexes were observed. Typically MRN can bind to DNA as monomer or dimer complexes, as multimers including 4 or more Rad50's and in DNA tethering complexes where MRN multimers are often found associating multiple DNA fragments. Multimerization favors DNA tethering which involves interactions of the coiled-coil Zn hook apexes (29, 30). Quantitation of DNA and complexes observed indicated that, as expected from the weak ability to form protein multimers, the MR(C2G) complex bound to DNA less efficiently. In addition, DNA tethering complexes were not observed with the C2G protein in the absence of ATP and were rare even with ATP present, consistent with the involvement of the Zn hook in tethering and its absence in this mutant. The difference between the number of DNA molecules bound by protein in either the absence or presence of ATP, comparing wild-type to the Rad50 C2G mutant, was statistically significant (*p* < 0.001 by chi square test). This confirmed the DNA binding deficiency, although the C2G mutant still showed increased binding to DNA in the presence of ATP.

##### Nbs1 Binds to MR in the Closed State

**FIGURE 7.**
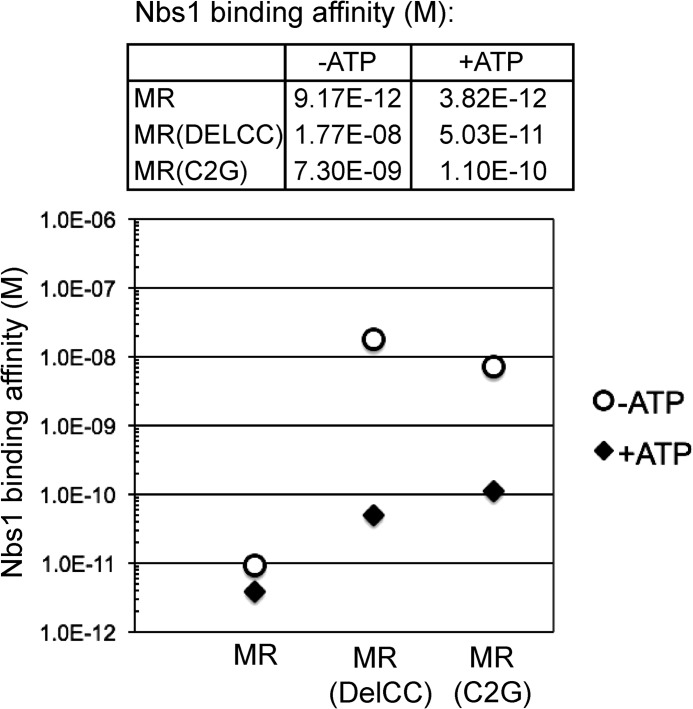
**ATP affects the association between MR and Nbs1 when the zinc hook or coiled-coils are disrupted.** Equilibrium dissociation constants (*M*) for Nbs1 binding to MR were measured using the KinExA system (see also supplemental Fig. S3). Nbs1 binding affinity for MR was analyzed with 1, 5, and 25 nm wild-type MR and the C2G and DelCC mutants, tested with titrations of 6.1 pm to 400 nm Nbs1 in 2-fold dilutions.

Nbs1 is critical for activation of ATM (2, 10), but little is known about the binding of Nbs1 to the MR complex, how the Rad50 coils and hook affect this interaction, and whether ATP regulates Nbs1 association. To investigate this, Nbs1 binding to MR was measured quantitatively using the KinExA system, which monitors the binding of a ligand to a protein of interest in solution (31). Wild-type Nbs1 at varying concentrations was mixed with MR complexes and free MR was measured by binding to Nbs1 immobilized on a capillary matrix, resulting in binding curves for the MR-Nbs1 interaction (Fig. 7 and supplemental Fig. S3). Results with the full-length MR complex showed that the affinity for Nbs1 is very high (9.17 pm) and that the affinity increases slightly in the presence of ATP (3.82 pm). However, a comparison of DelCC MR showed a very different result: the affinity for Nbs1 in the absence of ATP is reduced to 17.7 nm, while the affinity in the presence of ATP is 50.3 pm. A similar result was obtained with the C2G MR complex, where the affinity of MR for Nbs1 was ∼70-fold higher in the presence of ATP compared with the absence of ATP (Fig. 7). These observations show that ATP strongly influences the association of Nbs1 with MR, and suggests that Nbs1 binds to MR when it is in the closed, ATP-bound state.

Why is the effect of ATP on Nbs1 binding only observed in the absence of the zinc hook? In a recent study that employed site-directed crosslinking of the *P. furiosus* Rad50 protein, we found that the zinc hook connection allows full-length Rad50 to sample the closed state even in the absence of ATP.^5^ This clearly occurs with full-length *P. furiosus* Rad50 but was not observed with catalytic domains of PfRad50 expressed separately (equivalent to the DelCC human complex used here). Based on this result and the data from human Rad50 shown in the current study, we propose that the role of the zinc hook is primarily to provide a physical connection between the catalytic heads. In the absence of this connection, the association of the head domains into the closed structure is dependent on the presence of ATP but more importantly, on the absolute concentration of Rad50 head domains in solution. Constitutive dimerization of Rad50 via the zinc hook allows the formation of the closed structure to be independent of ATP and also independent of Rad50 concentration.

## DISCUSSION

Here we have investigated the enzymatic functions of the MRN complex and how these affect ATM kinase activity. Clearly the MRN complex plays a very important role in ATM function, particularly considering the similarity in clinical phenotypes of patients lacking ATM (A-T) compared with patients expressing hypomorphic alleles of *MRE11* or *RAD50* (A-T-like Disorder) or Nbs1 (Nijmegen Breakage Syndrome) (5). In addition, numerous studies using cell lines, extracts, and also purified systems have shown the importance of MRN for the activation of ATM by DNA DSBs (2, 3, 33–35). MRN has several distinct activities, however, not all of which are essential for the activation of ATM.

Using separate pools of ATP for ATM and MRN we show that ATP binding, but not hydrolysis, is essential for ATM activation via MRN. This suggests that MRN is in the “closed” configuration when bound to ATM on DNA (Fig. 1). This form of MRN is long-lived since the rate of ATP hydrolysis by Rad50 is slow, and is also the form of MRN that functions in DNA end tethering.^5^ Thus many of the important functions of the MRN complex take place with ATP bound but not hydrolyzed, consistent with the idea of ATP as a switch for Mre11/Rad50 conformation rather than an energy source for mechanical work.

During the course of these experiments we found that MRN function is strongly inhibited by ATP analogs containing modifications at the *N*6 position of the adenine base. This could be because the nitrogen at the *N*6 position is the only solvent-exposed part of the adenine base in the Rad50 ATP-bound conformation (36), so most of these analogs would have room to bind in the ATP-binding pocket. The *N*6-(furfuryl)-ATP compound was the only available *N*6-modified analog that did not show this toxicity.

Recent studies with the *P. furiosus* MR enzyme suggest that the nuclease activity of Mre11 is triggered by the change in conformation that occurs following ATP hydrolysis, from the “closed” back to the “open” state (see Fig. 1).^5^ The enzyme in the closed state does not exhibit nuclease activity, consistent with our observation in this study that Mre11 nuclease activity is also not required for ATM activation. Analysis of a knock-in allele of the Mre11(H129N) nuclease-deficient mutant in the mouse also indicated that nuclease activity of Mre11 was not required for ATM signaling, although there are clearly essential functions of the nuclease during development since the mouse showed early embryonic lethality (23).

### 

#### 

##### Roles of the Coiled-coil and the Zinc Hook

The coiled-coil domains in the Rad50 protein are conserved in all organisms although the length of the coils varies from ∼280 a.a. in *P. furiosus* Rad50 to ∼500 a.a. in human Rad50, equivalent to 30 to 50 nm per monomer when the coils are folded in an antiparallel manner (27). Mre11 binds to Rad50 at the base of the coiled-coil domain (37) but clearly the coils play a role that is independent from Mre11 binding since mutants that are deleted for most of the coils but still bind Mre11 are profoundly deficient in DNA repair and signaling *in vivo* in budding yeast, even when the hook is re-attached to the coil base to allow for Rad50 dimerization (38). Here we find that an equivalent mutant in human Rad50 (DelCC) fails to bind efficiently to DNA *in vitro*, showing that the coils actually contribute to MRN association with DNA. Consistent with this interpretation, we observed that the coils expressed and purified apart from the catalytic domains exhibit DNA binding activity. MR complexes have also been observed binding to DNA via the coiled-coil domain in SFM images.^4^ Collectively, these results suggest that the coiled-coil domain is not just a connector but actively participates in binding DNA and positioning the rest of the complex for optional function.

The zinc hook structure that connects two Rad50 monomers together has also been shown to be essential for MRN function in both yeast and mammalian cells (26, 39). In this study we find that the hook is important for ATM activation, although this deficiency can be suppressed by higher levels of the Rad50 hook mutants, unlike mutants in which the entire coiled-coil domain has been deleted. In this case the higher protein concentrations increase the probability of the head domains binding to each other, which facilitates both Nbs1 association and ATM activation. This suggests that the role of the hook is primarily to link the Rad50 monomers together, as evidenced by the ability of the heterologous FKBP homodimerization domain to substitute for the hook both *in vitro* (Fig. 5) and *in vivo* (28).

##### The Importance of the ATP-bound Closed State

The rate of ATP hydrolysis by Rad50 is very slow relative to other enzymes (∼0.1 molecules of ATP/minute) (11, 40).^5^ Functionally, the reason for this appears to be that many of the activities of MRN (DNA end binding, DNA tethering, and ATM activation) occur when MRN is in the closed state in which ATP is bound but not hydrolyzed (this study).^5^ Furthermore, the affinity of Nbs1 for the Mre11/Rad50(DelCC) complex is more than 300-fold higher when MR(DelCC) is bound to ATP, indicating that Nbs1 binds to MR when it is in the closed state.

Many enzymes require the formation of dimeric or multimeric complexes for optimal activity. Rad50 is an extreme example of this concept, in that the active site (for ATP hydrolysis) is composed of residues from two Rad50 molecules *in trans*. This situation would ordinarily ensure that catalytic activity would be strongly dependent on the affinity of the two domains for each other and on the absolute concentration of the domains in solution. However, the physical tether between Rad50 molecules in the form of the zinc hook makes the local concentration of the two catalytic domains extremely high, and allows the MRN complex to function optimally even when its absolute concentration in cells may vary widely between different cell types. Observations that the removal of the zinc hook from Rad50 *in vivo* results in nearly complete loss of function are consistent with this view (26, 28, 38, 39), and furthermore suggest that the absolute levels of Rad50 protein were not sufficiently high to drive formation of the closed complex when the monomers were not connected.

Schematic models of MRN function often portray Rad50 linking two broken DNA molecules, with the catalytic heads domains from one monomer bound to a distal site rather than associating with catalytic heads that are connected to it through the zinc hook. While it is difficult to discern the conformation of structures within the tethered complexes we have observed by SFM, our data suggest that MRN would be much more likely to form catalytic domain interactions within a single dimeric unit (as depicted in Fig. 1), particularly in the presence of ATP as illustrated previously (5, 29, 41). Association between head domains of a Rad50 dimer is also driven by Mre11 dimerization (6, 32). While it remains to be determined how ATP hydrolysis by Rad50 is regulated within cells, the physical characteristics of the protein support a model in which multimeric interactions are built upon the unit of a Rad50 dimer with the monomers associating within this unit at both the catalytic domains as well as the zinc hook and Mre11-binding interfaces.

In summary, our results show that the closed conformation of the human MRN complex is critical for the activation of the ATM kinase and that the structural components of the Rad50 protein that are important for this conformation are also important for the activation process. Other functions of MRN, including the stimulation of DNA end resection, are promoted by the conformational change from the closed to the open state that occurs during ATP hydrolysis but are not required for ATM activation. Thus there is a temporal sequence of DNA end tethering/ATM activation followed by DNA end resection that is orchestrated by MRN binding and hydrolysis of ATP.

## Footnotes

4 M. Mand, unpublished observations.

5 R. A. Deshpande, G. J. Williams, O. Limbo, R. S. Williams, J. Kuhnlein, J. H. Lee, S. Classen, G. Guenther, P. Russell, J. A. Tainer, and T. T. Paull (2013) Mre11-Rad50 Regulates DNA Tethering, End Resection, and ATM Checkpoint Signaling through ATP-driven RAD50 conformational changes, submitted publication.

## Footnotes

3 The abbreviations used are:

DSB: double-strand break
AMP-PNP: adenosine 5′-(β,γ-imino)triphosphate
ATM: ataxia telangiectasia-mutated
MRN: Mre11/Rad50/Nbs1
SFM: scanning force microscopy.
